# Polycystic ovary syndrome: What to say when asked about the chance of pregnancy

**DOI:** 10.1111/aogs.15149

**Published:** 2025-05-07

**Authors:** Laure Morin‐Papunen, Sari Pelkonen, Terhi Piltonen

**Affiliations:** ^1^ Research Unit of Clinical Medicine, Medical Research Center Oulu University of Oulu Oulu Finland; ^2^ Department of Obstetrics and Gynaecology Oulu University Hospital Oulu Finland

Polycystic ovary syndrome (PCOS) was described as early as 1844 by the French doctor Chereau and later by Stein and Leventhal in 1935. PCOS is the most common hormonal disorder in women and affects 11%–15% of the female population. The syndrome is lifelong, characterized by hyperandrogenism, chronic oligo‐anovulation, and high risk for several morbidities, and was long considered as an exclusively gynecological condition mainly affecting fertility.^1^, ^2^ This is true as PCOS covers about 30% of cases of infertility and 80% of cases with anovulatory infertility. Conversely, about 70%–80% of all women with PCOS have been estimated to suffer from infertility.^1^, ^2^ In addition to anovulation, an endometrial component seems to contribute to infertility and poor reproductive outcomes in affected women.^3^

It is generally accepted that PCOS affects fertility, and many women with PCOS are highly concerned about their reproductive capacity, often having a fear of remaining childless. However, results in general population studies have displayed some uncertainty of results on fecundity in PCOS, mainly due to differences in study population, length of follow‐up, and, in some studies, lack of adjustments for confounding factors, such as obesity, male infertility, or use of assisted reproductive technologies (ART).^4^, ^5^, ^6^, ^7^ In three population‐based studies,^4^, ^5^, ^6^ the overall fertility rate in women with PCOS was decreased,^4^, ^5^ and the women were more often nulliparous at an advanced age compared to other women.^6^ Moreover, the women ended up having their children older and eventually had fewer children compared to women without the syndrome, with a lower probability of having three or more children.^5^, ^6^ However, a Swedish register study^7^ and a population study from Finland^6^ showed that women with PCOS had a chance of having at least one child similar to that of other women and that their cumulative probability of childbirth was as high as in other women, especially if ART were available, as is the case in Nordic countries.

One explanation for the good reproductive capacity of women affected may be the increased ovarian reserve as shown by high serum anti‐Müllerian hormone (AMH) levels. However, PCOS characteristics, including menstrual cycles and systemic AMH levels, change with aging as the ovarian reserve and hormonal activity decrease, enabling a new, more optimal hormonal balance. Women with PCOS start gaining more regular cycles around the age of 35 years and may have an improved chance to conceive naturally.^8^ Conversely, this feature should be better recognized in women with PCOS over 40 years of age, as it may also lead to unplanned pregnancies especially if the women have considered themselves infertile their whole lives. To date, no data exist on induced abortions in PCOS.

Excess weight affects approximately 60%–70% of women with the syndrome, with large variation between individuals and geographic regions and ethnicity.^1^, ^2^ However, weight gain usually starts in childhood.^9^ Excess weight exacerbates insulin resistance with compensatory hyperinsulinemia and further development of the metabolic and reproductive disorders typical of PCOS. Obesity has direct effects both in the hypothalamic‐ovarian axis and in endometrial function and diminishes the chance of conceiving.^10^ Obesity also affects embryo quality.^11^ In some societies, a high body mass index (BMI) also limits access to publicly funded fertility treatment, thus promoting childlessness in this patient group.

Hence, the treatment of obesity is crucial to enable optimal fertility for affected women. Lifestyle (diet and exercise) is the first‐line management option and should always be a part of the PCOS management plan. In line, the new International PCOS Guidelines recommend pre‐pregnancy treatment with lifestyle and/or the insulin sensitizer metformin, or obesity‐targeting medications for all women with PCOS with BMI ≥ 25 kg/m^2^.^12^ Unfortunately, the results of these interventions are still modest, as most women with PCOS and obesity regain weight within 2 years after the intervention. Besides bariatric surgery, the emergence of new anti‐obesity glucagon‐like peptide 1 receptor agonists (GLP‐1 RAs) has brought great expectations in this patient group. The latest products, such as the GLP‐1 RAs and glucose‐dependent insulinotropic polypeptide (GIP) receptor dual agonist tirzepatide, have weight loss outcomes comparable to those achieved with bariatric surgery. The use of GLP‐1 RAs has been shown to induce significant improvements in insulin action, cardiovascular disease risk, weight reduction (including waist circumference), and reproductive function including increased pregnancy rates in overweight or obese PCOS women.^13^, ^14^ However, it must be noted that GLP‐1 receptor agonists are contraindicated during pregnancy. A recent meta‐analysis elaborated the risk for malformations in offspring born from pregnancies that were subjected to obesity medications, and no major concerns were raised regarding increased malformation rates.^15^ However, the weight gain after cessation of GLP‐1 receptor agonists in early pregnancy is of concern. In clinical practice, gastrointestinal disturbances, including nausea, vomiting, and diarrhea, are the most common side effects and reasons for medication discontinuation. In general, caution is warranted regarding the long‐term adverse effects of GLP‐1 RAs, including gastrointestinal disturbances, hypoglycemia, and effect on pancreatic health. A regular follow‐up with concomitant behavioral therapy to minimize weight regain has been shown to be successful and should always be implemented together with GLP‐1 RA medication. In addition, optimizing dosing strategies, closely monitoring patients, and tailoring treatment to individual needs are essential to maximizing their benefits while minimizing their drawbacks.^14^, ^16^ With the rapid rise of GLP‐1 RA use worldwide and the emergence of even more efficient preparations, their role in the treatment of obesity prior to pregnancy, including in PCOS, is expected to grow exponentially. Further research is warranted in women with PCOS to validate the efficacy, safety, and long‐term outcomes of these medications.

As an invasive treatment with small but real risks of complications, bariatric surgery is not the first‐line treatment option for fertility issues for women with PCOS and obesity. However, bariatric surgery was recently included in the recommendations of the international evidence‐based guidelines for PCOS^12^ and, in Finland, the national guideline to treat obesity has included PCOS diagnosis as one of the key factors supporting the decision on bariatric surgery. Supporting this, a recent, large randomized controlled study (RCT) (the “BAMBIBI‐trial”)^17^ assigned eighty women in a 1:1 ratio to either vertical sleeve gastrectomy or behavioral interventions and medical therapy (metformin or orlistat). The women were followed for 52 weeks. As expected, women in the surgical group experienced more spontaneous biochemically confirmed ovulations (2.5 times more often) and restoration of spontaneous menses, which suggests improvement in spontaneous fertility in these women. Indeed, larger, long‐lasting RCTs are required to further clarify the role of bariatric surgery in the treatment of reproductive disorders and pregnancy complications associated with PCOS as well as its safety regarding the health of the offspring.

In conclusion, what to say to a woman affected with PCOS when asked about the chance of pregnancy? First, as PCOS is a common condition with substantial effects on reproductive outcomes and is the most frequent cause of anovulatory infertility, the woman should be encouraged not to postpone pregnancy to be able to achieve the desired family size. Lifestyle intervention without stigmatizing the patient, promoting exercise alone or multicomponent management combining nutritional advice with exercise and behavioral strategies, together with metformin should be recommended. Information should also be given on the efficacy, but also on the short‐ and long‐term adverse effects of second‐line therapies, such as GLP1 RAs and bariatric surgery. On the other hand, healthcare providers should inform the patient that the good ovarian reserve in PCOS brings indisputable reproductive challenges but also advantages. Most importantly, with early and effective prevention and treatment of obesity and the use of ART, the patient should be informed that her chance of having at least one child is similar to that of women without the syndrome.

## Data Availability

Data sharing is not applicable to this article as no new data were created or analyzed in this study.

## References

[1] Joham AE , Norman R , Stener‐Victorin E , et al. Polycystic ovary syndrome. Lancet Diabetes Endocrinol. 2022;10:668‐680. 35934017 10.1016/S2213-8587(22)00163-2

[2] Stener‐Victorin E , Teede H , Norman R , et al. Polycystic ovary syndrome. Nat Rev Dis Primers. 2024;10:27. 38637590 10.1038/s41572-024-00511-3

[3] Palomba S , Piltonen TT , Giudice LC . Endometrial function in women with polycystic ovary syndrome: a comprehensive review. Hum Reprod Update. 2021;27:584‐618. 33302299 10.1093/humupd/dmaa051

[4] Koivunen R , Pouta A , Franks S , et al. Fecundability and spontaneous abortions in women with self‐reported oligo‐amenorrhea and/or hirsutism: Northern Finland Birth Cohort 1966 Study. Hum Reprod. 2008;23:2134‐2139. 18544581 10.1093/humrep/den136

[5] West S , Vähäsarja M , Bloigu A , et al. The impact of self‐reported oligo‐amenorrhea and hirsutism on fertility and lifetime reproductive success: results from the Northern Finland Birth Cohort 1966. Hum Reprod. 2014;29:628‐633. 24324025 10.1093/humrep/det437

[6] Forslund M , Teede H , Malin J , Tay CT , Loxton D , Joham AE . Fertility and age at childbirth in polycystic ovary syndrome: results from a longitudinal population‐based cohort study. Am J Obstet Gynecol. 2024;24:1135‐1139. 10.1016/j.ajog.2024.11.010 39547344

[7] Persson S , Elenis E , Turkmen S , Kramer MS , Yong EL , Sundström‐Poromaa I . Fecundity among women with polycystic ovary syndrome (PCOS)—a population‐based study. Hum Reprod. 2019;2019(34):2052‐2060. 10.1093/humrep/dez159 31504532

[8] Van Keizerswaard J , Dietz de Loos A , Louwers Y , Laven JSE . Changes in individual polycystic ovary syndrome phenotypical characteristics over time: a long‐term follow‐up study. Fertil Steril. 2022;117:1059‐1066. 35219451 10.1016/j.fertnstert.2022.01.014

[9] Koivuaho E , Laru J , Ojaniemi M , et al. Age at adiposity rebound in childhood is associated with PCOS diagnosis and obesity in adulthood‐longitudinal analysis of BMI data from birth to age 46 in cases of PCOS. Int J Obes. 2019;43:1370‐1379. 10.1038/s41366-019-0318-z PMC6760596 30718819

[10] Luyckx L , Myllykangas M , Saarela U , et al. Prenatally androgenized PCOS mice have ovary‐independent uterine dysfunction and placental inflammation aggravated by high‐fat diet. Sci Adv. in press; 2025. 10.1126/sciadv.adu3699 PMC12063661 40344073

[11] Broughton DE , Moley KH . Obesity and female infertility: potential mediators of obesity's impact. Fertil Steril. 2017;107:840‐847. 28292619 10.1016/j.fertnstert.2017.01.017

[12] Teede HJ , Tay CT , Laven JJE , et al. Recommendations from the 2023 international evidence‐based guideline for the assessment and management of polycystic ovary syndrome. Eur J Endocrinol. 2023;189:G43‐G64. 37580861 10.1093/ejendo/lvad096

[13] Cena H , Chiovato L , Nappi RE , et al. Obesity, polycystic ovary syndrome, and infertility: a new avenue for GLP‐1 receptor agonists. J Clin Endocrinol Metab. 2020;105:e2695‐e2709. doi:10.1210/clinem/dgaa285 32442310 PMC7457958

[14] Fadel LP , Thao G , Chitre T , et al. A system‐based review on effects of glucagon‐like peptide‐1 receptor agonists: benefits vs risks. Cureus. 2025;17:e78575. 40062098 10.7759/cureus.78575 PMC11888820

[15] Cesta CE , Bateman R , Chodick G , et al. Safety of GLP‐1 receptor agonists and other second‐line antidiabetics in early pregnancy. JAMA Intern Med. 2024;184:144‐152. 38079178 10.1001/jamainternmed.2023.6663 PMC10714281

[16] Frangie Machado M , Shunk T , Hansen G , et al. Clinical effects of glucagon‐like peptide‐1 agonist use for weight loss in women with polycystic ovary syndrome: a scoping review. Cureus. 2024;16:e66691. doi:10.7759/cureus.66691 39262529 PMC11389649

[17] Samarasinghe S , Leca B , Alabdulkader S , et al. Bariatric surgery for spontaneous ovulation in women living with polycystic ovary syndrome: the BAMBINI multicentre, open‐label, randomised controlled trial. Lancet. 2024;403:2489‐2503. 38782004 10.1016/S0140-6736(24)00538-5

